# Supplementary material for: The causal role of circulating inflammatory markers in osteoporosis: a bidirectional Mendelian randomized study
Source: Front Immunol. 2024 Jul 18;15:1412298. doi: 10.3389/fimmu.2024.1412298 (PMC11291241; doi:10.3389/fimmu.2024.1412298)
Supplement: Supplementary file 1 [file DataSheet_1.zip › supplementary material/OSTEOPOROSIS-CIM/GCST90274767.tsv.gz_leave_one_out.pdf]

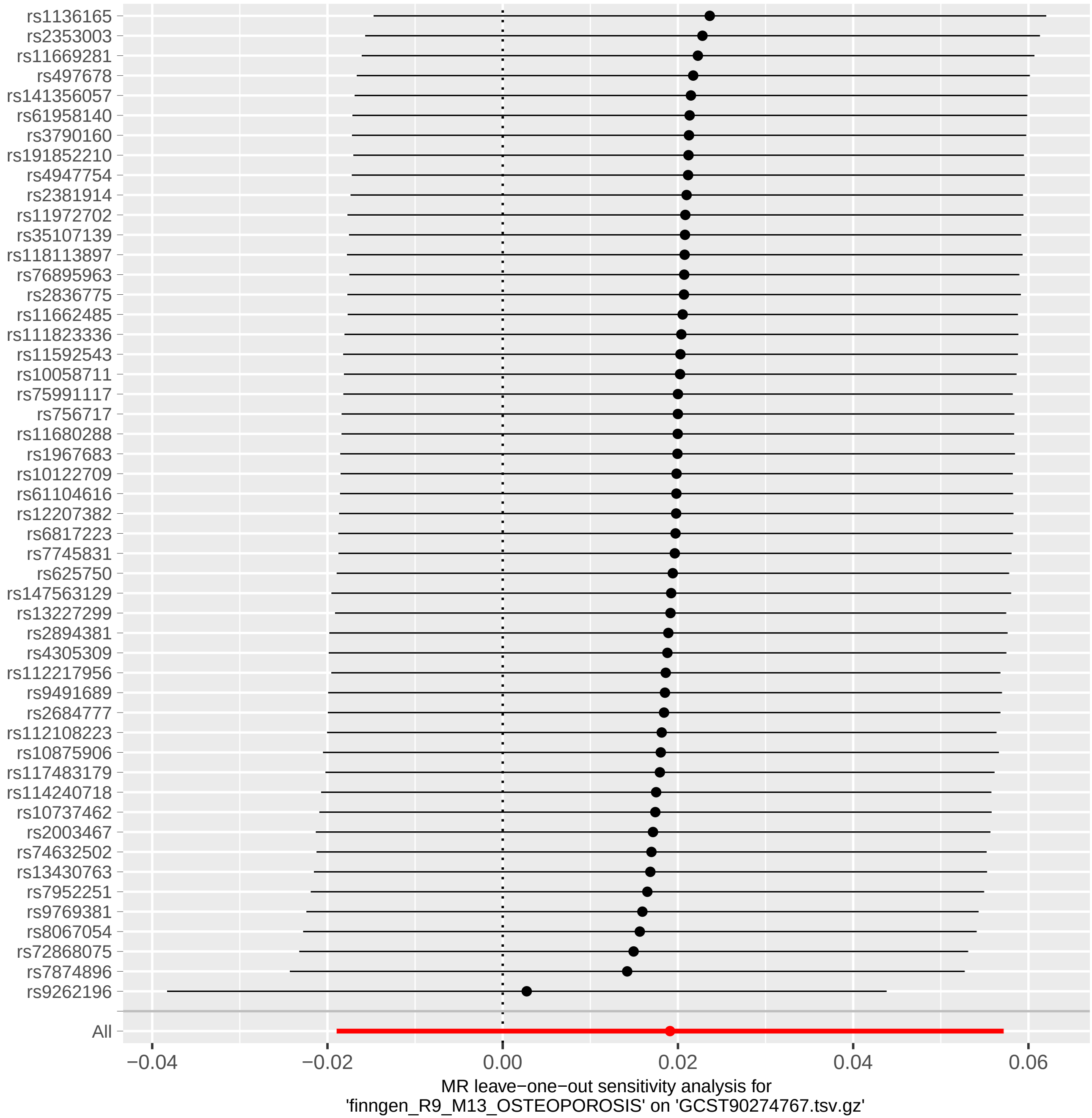
rs1136165
rs2353003
rs11669281
rs497678
rs141356057
rs61958140
rs3790160
rs191852210
rs4947754
rs2381914
rs11972702
rs35107139
rs118113897
rs76895963
rs2836775
rs11662485
rs111823336
rs11592543
rs10058711
rs75991117
rs756717
rs11680288
rs1967683
rs10122709
rs61104616
rs12207382
rs6817223
rs7745831
rs625750
rs147563129
rs13227299
rs2894381
rs4305309
rs112217956
rs9491689
rs2684777
rs112108223
rs10875906
rs117483179
rs114240718
rs10737462
rs2003467
rs74632502
rs13430763
rs7952251
rs9769381
rs8067054
rs72868075
rs7874896
rs9262196
All
−0.04
−0.02
0.00
0.02
0.04
0.06
MR leave−one−out sensitivity analysis for
'finngen_R9_M13_OSTEOPOROSIS' on 'GCST90274767.tsv.gz'
